# Portal vein reconstruction reduces textbook outcome achievement following radical resection for hilar cholangiocarcinoma

**DOI:** 10.3389/fonc.2026.1748250

**Published:** 2026-02-27

**Authors:** Jialin Li, Xinchun Li, Yanmin Chen, Yulin Li, Ting Hu, Yang Liu

**Affiliations:** 1 Department of General Surgery, Changde Hospital, Xiangya School of Medicine, Central South University, Changde, China; 2 Department of Hepatobiliary Surgery, Southwest Hospital, Army Medical University, Chongqing, China

**Keywords:** hilar cholangiocarcinoma, portal vein reconstruction, predictors, prognosis, textbook outcome

## Abstract

**Background:**

Hilar cholangiocarcinoma (HCCA) remains a surgically challenging malignancy, often requiring major hepatectomy with vascular resection and reconstruction to achieve R0 resection. Portal vein reconstruction (PVR) enables radical resection in patients with vascular invasion, while its impact on surgical quality, measured using textbook outcome (TO), remains unclear.

**Methods:**

A total of 317 HCCA patients who underwent R0 resection at a single tertiary medical center were retrospectively analyzed. In this study, TO was defined as the absence of 90-day mortality, readmission within 90 days, post-operative severe comorbidities, post-operative bile leak, post-operative liver failure, and intraoperative severe incidents. Epidemiological characteristics, pre-operative examination results, intraoperative features, post-operative comorbidities, and survival were compared between the PVR (n = 62) and non-PVR (n = 255) groups. The predictors of TO were evaluated using univariate and multivariate logistic regression analyses. The Kaplan–Meier curves were used to assess overall survival (OS) and relapse-free survival (RFS).

**Results:**

In this study, 113 of 317 patients (35.65%) achieved TO. TO rates were significantly lower in the PVR group (20.97%) compared with the non-PVR group (39.22%, p = 0.007). Patients with PVR had higher rates of post-operative infection (73.8% *vs*. 53.6%, p = 0.004), bile leakage (32.8% *vs*. 13.5%, p < 0.001), and liver failure (8.2% *vs*. 2.0%, p = 0.038). After univariate and multivariate analyses, PVR was identified as an independent negative predictor of TO (OR = 0.48, p = 0.046). Furthermore, the Kaplan–Meier analysis indicated significantly worse OS and RFS in both the non-TO and PVR groups (all p < 0.001).

**Conclusions:**

PVR is significantly associated with reduced TO achievement and impaired long-term outcomes following R0 resection for HCCA patients. Although PVR remains a necessary approach to achieve curative resection in advanced cases, its impact highlights the need for careful patient selection and optimization of peri-operative management to improve the clinical outcomes of these patients.

## Introduction

Hilar cholangiocarcinoma (HCCA) is a rare but highly aggressive malignant tumor arising from the hilar bile duct, accounting for approximately 50% of all cholangiocarcinomas (1). Surgical resection remains the only curative treatment method, while the surgical procedure is technically demanding due to the tumor’s anatomical proximity to major vascular structures, particularly the portal vein and hepatic artery (2–4). Achieving an R0 margin of HCCA frequently requires extended hepatectomy combined with vascular resection and reconstruction (5).

Portal vein reconstruction (PVR) is increasingly performed to achieve margin-negative resection in HCCA patients with vascular invasion (6). However, the prognostic benefits of PVR remain controversial (7). Although it may expand resectability, the complexity of the procedure is also associated with longer operative time, increased blood loss, and higher post-operative morbidities. Previous studies have reported conflicting results regarding whether PVR confers a survival advantage or instead compromises surgical safety and long-term outcomes (8).

In recent years, the concept of textbook outcome (TO) has been introduced as a composite quality indicator in surgical oncology (9). TO reflects the achievement of an ideal peri-operative condition, consisting of the absence of major complications, readmission, and early post-operative mortality (10). Unlike a single endpoint, TO provides a comprehensive measure of surgical quality that can be meaningfully compared across procedures and institutions (11, 12). To date, there is limited evidence concentrating on the rate of TO achievement in HCCA and the influence of PVR on TO after radical resection (13).

In this study, we retrospectively analyzed 317 HCCA patients undergoing R0 resection, with the aim of comparing peri-operative outcomes and TO achievement rate between the PVR and non-PVR groups. Furthermore, we evaluated the potential predictors of TO in HCCA patients.

## Methods

### Patients and study design

This retrospective cohort study included patients who underwent R0 resection at a tertiary hospital between January 2014 and June 2020. This study was approved by the Ethics Committee of Southwest Hospital. Eligible patients had histologically confirmed HCCA and achieved R0 resection, defined as microscopically negative margins. Exclusion criteria included R1/R2 resection, pre-operative chemotherapy, incomplete clinical data, or lost to follow-up within 90 days after surgery. Patients were stratified into two groups according to whether PVR was performed (PVR *vs*. non-PVR).

### Data collection

The epidemiological, laboratory, imaging, operative, and pathological data were collected from electronic medical records. Collected variables included demographic characteristics, comorbidities, biochemical examination results, operative details (including extent of hepatectomy, vascular invasion, operation time, transfusion, and blood loss), post-operative complications, length of hospital stay, and total hospitalization costs. Tumor characteristics, including tumor size, Bismuth classification, differentiation grade, lymph node metastasis, and pathologically endoscopic invasion, were also included. The large-scale liver resection in this study was defined as a major hepatectomy [often a right/left or extended (tri-segment) hepatectomy], routinely combined with en bloc caudate lobe resection, extrahepatic bile duct excision, and regional lymphadenectomy, with an anticipated liver resection volume ≥50%. The preoperative biliary drainage methods in the study included percutaneous transhepatic biliary drainage (PTBD) and endoscopic nasobiliary drainage (ENBD).

### Definition of textbook outcome

**Figure 1:**
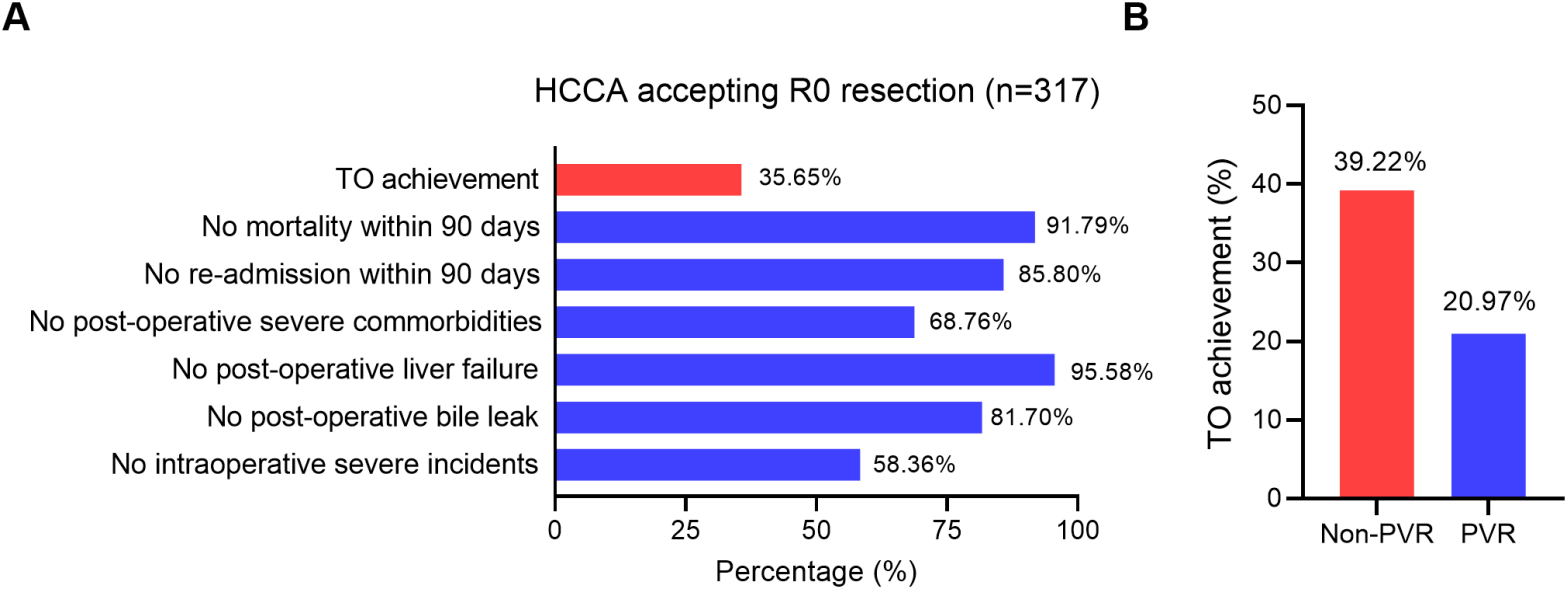
Textbook outcome evaluations of HCCA patients accepting radical resection. **(A)** Details of textbook outcome. **(B)** Percentage of HCCA patients with PVR and non-PVR. HCCA, hilar cholangiocarcinoma; PVR, portal vein reconstruction.

TO was defined as the simultaneous fulfillment of several peri-operative quality criteria. In this study, for patients undergoing R0 resection for hilar cholangiocarcinoma, TO required the absence of in-hospital or 90-day mortality, no readmission within 90 days, no severe post-operative complications classified as Clavien–Dindo grade III or higher, no intraoperative severe incidents (defined using the Oslo classification) (14), and no occurrence of post-operative grade B/C bile leakage and liver failure according to the definitions of the International Study Group of Liver Surgery (15, 16) (**Figure 1A**). Only patients meeting all criteria were considered to have achieved TO, while failure in any single domain was classified as non-TO.

### Surgery and follow-up

The decision to perform PVR was based on preoperative imaging and intraoperative findings of portal vein involvement by the chief surgeon. Reconstruction techniques included end-to-end anastomosis or autologous vein grafting. Patients were followed up every 3–6 months for the first 2 years and annually thereafter. After R0 resection, we recommended that patients take capecitabine for up to 8 months. Overall survival (OS) was defined as the interval between surgery and death from any cause or last follow-up. Relapse-free survival (RFS) was defined as the time from surgery to tumor recurrence or last follow-up.

### Statistical analysis

Continuous variables were expressed as mean ± standard deviation or median with interquartile range and compared using Student’s t-test or the Mann–Whitney U test. Categorical variables were compared using the χ^2^ test or Fisher’s exact test. Logistic regression analyses were performed to identify independent predictors and to assess whether PVR remained independently associated with textbook outcome after adjustment for other clinically relevant variables within a uniform R0 cohort. Variables with p < 0.05 in univariate analysis were entered into a multivariate regression model. Survival curves for OS and RFS were estimated using the Kaplan–Meier method and compared with the log-rank test. A two-tailed p < 0.05 was considered statistically significant. Analysis was performed using STATA version 14.0.

## Results

### Patient characteristics

A total of 317 patients who underwent R0 resection for hilar cholangiocarcinoma were included, comprising 62 patients in the PVR group and 255 in the non-PVR group. As shown in **Table 1**, baseline demographic and clinical characteristics were largely comparable (p > 0.05) between groups, including age, sex, comorbidities, and hematologic parameters. However, pre-operative liver function tests revealed significantly higher alanine aminotransferase (ALT) (194.4 *vs*. 149.4 U/L, p = 0.037) and alkaline phosphatase (ALP) (632.2 *vs*. 522.5 U/L, p = 0.040) levels in the PVR group.

**Table 1:** Basic characteristics of HCCA patients with PVR and non-PVR.

Variables	Total	Non-PVR	PVR	P
	(n = 317)	(n = 255)	(n = 62)	
Age (years)	58.18 ± 10.07	58.40 ± 10.26	57.29 ± 9.27	0.439
BMI (kg/m^2^)	22.14 ± 2.85	22.03 ± 2.98	22.57 ± 2.26	0.144
Diabetes, n (%)	24 (7.59)	19 (7.48)	5 (8.06)	0.876
Hypertension, n (%)	41 (12.97)	33 (12.99)	8 (12.90)	0.985
CHD, n (%)	7 (2.22)	5 (1.97)	2 (3.23)	0.546
HBV, n (%)	22 (7.77)	21 (9.29)	1 (1.75)	0.105
Liver cirrhosis, n (%)	21 (6.62)	15 (5.88)	6 (9.68)	0.428
WBC (×10^9^/L)	6.82 ± 2.43	6.74 ± 2.39	7.16 ± 2.61	0.235
HB (g/L)	124.04 ± 25.31	123.16 ± 27.02	127.53 ± 16.65	0.237
PLT (×10^9^/L)	248.97 ± 91.20	249.00 ± 89.51	248.85 ± 98.40	0.991
ALT (U/L)	158.53 ± 149.22	149.43 ± 150.42	194.44 ± 139.89	0.037
AST (U/L)	141.27 ± 140.55	134.55 ± 136.29	168.05 ± 154.73	0.105
ALP (U/L)	544.67 ± 368.89	522.52 ± 372.27	632.15 ± 344.38	0.04
γ-GGT (U/L)	748.43 ± 1,240.47	720.40 ± 1,341.20	860.99 ± 700.70	0.437
Albumin (g/L)	37.93 ± 5.08	37.75 ± 5.17	38.63 ± 4.70	0.232
Prealbumin (mg/L)	132.01 ± 63.81	132.88 ± 60.86	128.92 ± 73.99	0.702
Highest bilirubin (μmol/L)	216.50 ± 152.47	210.71 ± 153.33	239.36 ± 148.05	0.194
Preoperational bilirubin (μmol/L)	197.60 ± 142.66	190.99 ± 141.79	223.72 ± 144.25	0.113
Glucose (mmol/L)	25.85 ± 315.48	30.94 ± 353.24	5.79 ± 1.30	0.612
Na (mmol/L)	137.98 ± 8.63	137.83 ± 9.57	138.54 ± 2.76	0.585
K (mmol/L)	4.40 ± 8.17	4.53 ± 9.14	3.90 ± 0.39	0.606
Ca (mmol/L)	2.30 ± 0.13	2.30 ± 0.13	2.31 ± 0.13	0.529
Creatinine (μmol/L)	64.50 ± 19.51	63.46 ± 18.54	68.58 ± 22.63	0.074
BUN (μmol/L)	5.89 ± 10.73	6.05 ± 11.94	5.27 ± 3.08	0.631
PT (s)	16.50 ± 71.27	17.77 ± 79.63	11.42 ± 1.23	0.544
AFP (ng/mL)	5.39 ± 5.20	5.12 ± 4.79	6.42 ± 6.54	0.095
PTBD, n (%)	121 (38.17)	96 (37.65)	25 (40.32)	0.697
ENBD, n (%)	109 (34.38)	88 (34.51)	21 (33.87)	0.924
Adjuvant chemotherapy, n (%)	248 (78.25)	195 (76.47)	53 (85.48)	0.123

HCCA, hilar cholangiocarcinoma; PVR, portal vein reconstruction; CHD, coronary heart disease; WBC, white blood cell; HB, hemoglobin; PLT, platelet; ALT, alanine aminotransferase; AST, aspartate aminotransferase; ALP, alkaline phosphatase; γ-GGT, gamma-glutamyltransferase; PT, prothrombin time; AFP, alpha-fetoprotein; PTBD, percutaneous transhepatic biliary drainage; ENBD, endoscopic nasobiliary drainage; HBV, Hepatitis B virus; BUN, Blood Urea Nitrogen.

### Operative findings

Operative details are summarized in **Table 2**. The PVR group had a significantly higher proportion of Bismuth–Corlette type III/IV (90.3% *vs*. 66.9%, p < 0.001) compared with the non-PVR group. Portal vein invasion (95.2% *vs*. 16.9%, p < 0.001), hepatic artery invasion (53.2% *vs*. 16.9%, p < 0.001), and hepatic artery reconstruction (22.58% *vs*. 5.49%, p < 0.001) were also more prevalent in the PVR group than the non-PVR group. These findings indicate that those patients requiring PVR had more advanced disease and underwent more complex operations.

**Table 2:** Operative characteristics of HCCA patients with PVR and non-PVR.

Variables	Total	Non-PVR	PVR	P
	(n = 317)	(n = 255)	(n = 62)	
Blood loss (mL)	771.32 ± 604.98	744.06 ± 575.95	887.27 ± 709.34	0.102
Tumor diameter (cm)	5.38 ± 18.36	5.32 ± 19.32	5.60 ± 14.06	0.918
Bismuth ≥ III, n (%)	224 (71.57)	168 (66.93)	56 (90.32)	<0.001
Large-scale liver resection, n (%)	197 (69.61)	144 (64.57)	53 (88.33)	<0.001
Blood transfusion, n (%)	192 (61.34)	151 (59.92)	41 (67.21)	0.294
Portal vein invasion, n (%)	102 (32.18)	43 (16.86)	59 (95.16)	<0.001
Hepatic artery invasion, n (%)	76 (23.97)	43 (16.86)	33 (53.23)	<0.001
Hepatic artery reconstruction, n (%)	28 (8.83)	14 (5.49)	14 (22.58)	<0.001
Poor differentiation, n (%)	61 (19.93)	48 (19.59)	13 (21.31)	0.764
Lymph node metastasis, n (%)	115 (36.51)	86 (33.99)	29 (46.77)	0.061
Ki67 (%)	21.75 ± 15.41	22.53 ± 15.40	18.89 ± 15.31	0.198
Endoscopic vascular invasion, n (%)	31 (9.84)	23 (9.06)	8 (13.11)	0.339
Endoscopic nerve invasion, n (%)	134 (42.81)	110 (43.65)	24 (39.34)	0.542

HCCA, hilar cholangiocarcinoma; PVR, portal vein reconstruction.

### Post-operative outcomes and complications

In this study, the overall rate of TO achievement was 35.65% (113/317) (**Figure 1A**). Patients in the PVR group had a significantly lower TO rate than those in the non-PVR group (20.97% *vs*. 39.22%, p = 0.007; **Figure 1B**). Post-operative complication profiles differed notably between groups: patients who underwent PVR experienced higher rates of fever (54.10% *vs*. 35.32%, p = 0.002), bile leakage (38.71% *vs*. 13.33%, p = 0.001), infection (73.77% *vs*. 53.57%, p = 0.004), and post-hepatectomy liver failure (9.68% *vs*. 3.14%, p = 0.025) compared with the non-PVR group (**Table 3**). Furthermore, as a key component of the TO, the 90-day readmission rate was significantly higher in the PVR group compared to the non-PVR group (24.19% *vs*. 11.76%, p = 0.012). Furthermore, the 90-day mortality rate was also slightly higher in the PVR group (11.29% *vs*. 7.45%), although not statistically significant (p > 0.05).

**Table 3:** Post-operative complications between HCCA patients with PVR and non-PVR.

Variables	Total	Non-PVR	PVR	P
	(n = 317)	(n = 255)	(n = 62)	
Clavien–Dindo grade ≥ III	40 (14.83)	29 (11.37)	11 (17.74)	0.176
Fever, n (%)	122 (38.98)	89 (35.32)	33 (54.10)	0.002
Bile leakage, n (%)	58 (18.30)	34 (13.33)	24 (38.71)	0.001
Infection, n (%)	180 (57.51)	135 (53.57)	45 (73.77)	0.004
Cholangitis, n (%)	17 (5.45)	12 (4.78)	5 (8.20)	0.459
Bowel leakage, n (%)	4 (1.28)	4 (1.59)	0 (0.00)	1
Hydrothorax diameter > 3 cm, n (%)	63 (21.28)	49 (20.76)	14 (23.33)	0.664
Abdominal puncture drainage, n (%)	50 (16.03)	41 (16.33)	9 (14.75)	0.763
Post-operative hemorrhage, n (%)	115 (38.33)	89 (37.08)	26 (43.33)	0.373
Post-hepatectomy liver failure, n (%)	14 (4.42)	8 (3.14)	6 (9.68)	0.025
Hospital stays (days)	24.40 ± 13.23	23.94 ± 13.39	26.28 ± 12.50	0.217
90-day mortality, n (%)	26 (8.20)	19 (7.45)	7 (11.29)	0.323
Readmission within 90 days, n (%)	45 (14.20)	30 (11.76)	15 (24.19)	0.012

HCCA, hilar cholangiocarcinoma; PVR, portal vein reconstruction.

### Predictors of textbook outcome

Furthermore, univariate and multivariate logistic regression analyses were used to identify potential predictors of TO achievement. As shown in **Table 4**, liver cirrhosis (OR = 0.27, p = 0.037), high total bilirubin (OR = 0.27, p = 0.037), large-scale liver resection (OR = 0.59, p = 0.049), and PVR (OR = 0.41, p = 0.008) were negative predictors of TO in the univariate logistic regression. These four factors were selected to enter multivariate logistic regression, while the result confirmed that the PVR was an independent factor associated with reduced TO achievement (OR = 0.48, p = 0.046).

**Table 4:** Univariate and multivariate logistic regression analyses for predictors of TO.

Variables	Univariate analysis		Multivariate analysis	
	OR (95% CI)	p	OR (95% CI)	P
Diabetes (yes *vs*. no)	1.03 (0.44–2.42)	0.942		
Hypertension (yes *vs*. no)	1.20 (0.61–2.36)	0.596		
CHD (yes *vs*. no)	1.38 (0.30–6.29)	0.675		
HBV (yes *vs*. no)	1.16 (0.48–2.79)	0.738		
Liver cirrhosis (yes *vs*. no)	0.27 (0.08–0.92)	0.037	0.38 (0.11~ 1.37)	0.139
ASA (III/IV *vs*. I/II)	0.88 (0.55–1.40)	0.58		
Preoperative comorbidities (yes *vs*. no)	0.94 (0.56–1.55)	0.795		
Age (≥60 *vs*. <60)	1.06 (0.67–1.67)	0.817		
Albumin (≥35 *vs*. <35)	0.99 (0.58–1.69)	0.965		
ALT (≥42 *vs*. <42)	0.84 (0.45–1.57)	0.589		
Total bilirubin (≥42 *vs*. <42)	0.51 (0.28–0.94)	0.029	0.62 (0.32–1.19)	0.151
Large-scale liver resection (yes *vs*. no)	0.59 (0.35–0.99)	0.049	0.70 (0.40–1.23)	0.216
PVR (yes *vs*. no)	0.41 (0.21–0.80)	0.008	0.48 (0.24–0.99)	0.046
HAR (yes *vs*. no)	0.57 (0.24–1.39)	0.218		
PVR and HAR (yes *vs*. no)	0.71 (0.22–2.32)	0.573		
Poor differentiation (yes *vs*. no)	0.89 (0.49–1.62)	0.705		
Lymph node metastasis (yes *vs*. no)	0.66 (0.41–1.07)	0.091		
PTBD (yes *vs*. no)	0.74 (0.46–1.20)	0.228		
Bismuth (III/IV *vs*. I/II)	0.84 (0.51–1.40)	0.506		

TO, textbook outcome; CHD, coronary heart disease; ALT, alanine aminotransferase; PVR, portal vein reconstruction; PTBD, percutaneous transhepatic biliary drainage; ASA, American Society of Anesthesiologists Physical Status Classification System; HAR, Hepatic artery reconstruction.

### Survival analysis

**Figure 2:**
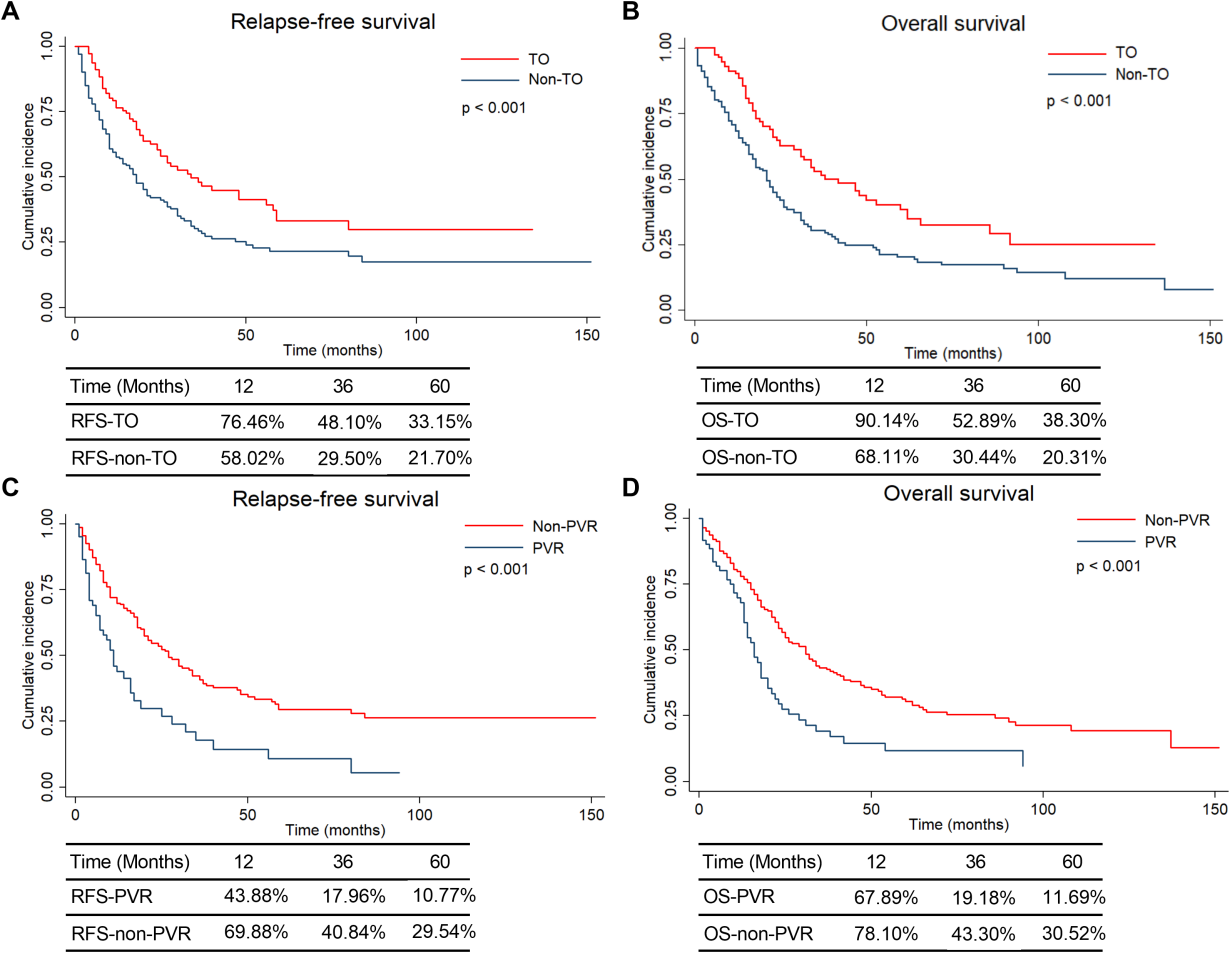
Survival curves of HCCA patients with TO, Non-TO, PVR and Non-PVR. **(A)** Relapse-free survival and relevant survival rate of HCCA patients with TO and Non-TO; **(B)** Overall survival and relevant survival rate of HCCA patients with TO and Non-TO; **(C)** Relapse-free survival and relevant survival rate of HCCA patients accepting PVR and Non-PVR; **(D)** Overall survival and relevant survival rate of HCCA patients accepting PVR and Non-PVR.

The Kaplan–Meier analysis demonstrated that patients achieving TO had significantly longer OS and RFS compared to those with non-TO (both p < 0.001). Similarly, the PVR group showed significantly worse OS and RFS compared with the non-PVR group (both p < 0.001; **Figure 2**). The actual 1-year, 3-year, and 5-year survival rates of each group are also shown in **Figure 2**. These results highlighted the prognostic importance of TO and suggest that PVR may negatively influence long-term survival, although it was necessary for radical resection in advanced cases.

## Discussion

In this study, we demonstrated that PVR was significantly associated with reduced TO achievement and worse long-term survival. Logistic regression analysis confirmed PVR as an independent negative predictor of TO. Furthermore, the Kaplan–Meier curves showed that both the non-TO and PVR groups experienced significantly poorer overall OS and RFS.

The concept of TO provides a multi-dimensional assessment of peri-operative quality, moving beyond single endpoints such as morbidity or mortality (17, 18). In this study, TO achievement correlated strongly with improved OS and RFS, reinforcing its utility as a surrogate marker for prognostic outcomes in HCCA surgery. This finding aligns with prior work in hepatobiliary and oncologic surgery, where TO has emerged as a benchmark to compare surgical quality across institutions and techniques (13, 19). Incorporating TO into clinical practice may enhance patient counseling, facilitate quality improvement initiatives, and serve as a meaningful endpoint in future surgical trials (20).

PVR was necessary in approximately 20% of our cohort, reflecting its role in enabling radical resection for HCCA with vascular involvement (21). However, patients requiring PVR presented with more advanced tumors and underwent more complex procedures, as evidenced by higher rates of large-scale hepatectomy and concurrent arterial invasion (22). These factors likely contributed to the higher incidence of severe complications and lower TO rates. Importantly, the adverse effect of PVR on long-term survival highlights the need for careful patient selection and meticulous peri-operative management. While PVR extends resectability, its prognostic disadvantage raises questions about balancing surgical radicality with post-operative prognosis (23–25).

Our findings suggest that achieving TO may be a primary goal in HCCA surgery, as it directly influences survival (11). Strategies to improve TO rates in PVR patients may include pre-operative optimization (nutritional support, biliary drainage, or neoadjuvant therapy), refinement of vascular reconstruction techniques, and enhanced post-operative management (26). Additionally, integrating TO into multidisciplinary decision-making could help identify patients who are most likely to benefit from aggressive surgery versus those who may be better served by alternative approaches.

However, this study also has several limitations. First, its retrospective single-center design includes potential selection bias, and the sample size of the PVR group was relatively small. Second, variations in surgical techniques and peri-operative management over the long study period (2008–2022) may also have influenced outcomes. Moreover, although multivariate analysis was performed, unmeasured confounders cannot be excluded. Prospective multicenter studies are needed to validate our findings and further refine the role of PVR in the management of HCCA.

## Conclusion

TO after radical resection in HCCA patients represents a multi-dimensional assessment indicator for clinical prognosis and long-term survival. PVR is an independent predictor of reduced TO of HCCA patients.

## Data Availability

The raw data supporting the conclusions of this article will be made available by the authors, without undue reservation.

## Glossary

HCCA: hilar cholangiocarcinoma
TO: textbook outcome
PVR: portal vein reconstruction
CHD: coronary heart disease
WBC: white blood cell
HB: hemoglobin
PLT: platelet
ALT: alanine aminotransferase
AST: aspartate aminotransferase
γ-GGT: gamma-glutamyltransferase
ALP: alkaline phosphatase
PT: prothrombin time
AFP: alpha-fetoprotein
OS: overall survival
RFS: relapse-free survival
